# A Leech in the Larynx

**Published:** 1890-03

**Authors:** 


### A Leech in the Larynx.

Prof. F. Massei, in an interesting article on foreign bodies in the air passages, in the *Archiv. di Laryngolog.*, reported among other noteworthy cases that of a man who had suffered since a fortnight with profuse hæmopteses. He came home exceedingly weak, and dated the beginning of his trouble from his swallowing some water from the water casks of a vessel upon which he was sailing. At the laryngological examination a leech was found, attached to the right ary-epiglottidean ligament. It was at once removed, and the symptoms relieved.—*Intern. Journ. of Surgery*, Jan., 1890.

ACCORDING to the statement of the Registrar of the New York Board of Health, the greater ratio of deaths occur in old houses, modern houses being more conductive to health.

